# Determination of car seat contact area for personalised thermal sensation modelling

**DOI:** 10.1371/journal.pone.0208599

**Published:** 2018-12-11

**Authors:** Miloš Fojtlín, Agnes Psikuta, Róbert Toma, Jan Fišer, Miroslav Jícha

**Affiliations:** 1 Laboratory for Biomimetic Membranes and Textiles, Empa Swiss Federal Laboratories for Material Science and Technology, St. Gallen, Switzerland; 2 Department of Thermodynamics and Environmental Engineering, Energy Institute, Faculty of mechanical engineering, Brno University of Technology, Brno, Czechia; US Army Research Institute of Environmental Medicine, UNITED STATES

## Abstract

A lot of daily activities are conducted in a sedentary posture. This includes a thermal interaction between the human and the seat that has implications on thermal perception and comfort. These interactions are investigated by simulating heat and mass transfer, thus, reducing a need for costly and time demanding subject studies. However, it is not clear, from the available literature, what portion of the body surface area is actually affected by the seat with respect to human anthropometry. The aim of this study was to develop a predicting function of the seat contact area based on anthropometric parameters. The results showed strong linear correlation between the contact area obtained by printing a body silhouette on paper placed at the seat and body weight, height, body surface area, and body mass index. The body surface area and the body weight were identified as the best predictors for the contact area.

## Introduction

Western lifestyle is bound to seated posture during work, travelling or leisure time with more than 50% of wake time spent sitting [1,2]. For this reason, the effects of sitting on health and comfort have been investigated in detail [3–6]. Moreover, the state of comfort is closely related to vigilance and well-being that is needed to ensure productive conditions at a workplace or safe and enjoyable experience while driving or travelling [7–9].

Various aspects of comfort and discomfort in an office environment in sitting were examined by Zhang et al. [10]. Office features with the highest importance to office workers were ascribed to chair comfort (12% of responses) and effects of an ambient microclimate (11% of responses). Moreover, similar conclusions were found in an airplane passenger comfort study, where again the best predictor for general comfort on the flight was found the seat comfort [9].

One of the aspects of comfort is thermal comfort that expresses satisfaction with the thermal environment. In addition to a clothing resistance, the seat creates thermal and water vapour barrier at the contact body parts. As a result, the seat’s high thermal insulation and low water vapour permeability affect local microclimate at the body surface that may lead, under certain circumstances, to a local discomfort, sweating, and perception of wetness [11–13].

A very specific application of seats are the ones used in vehicular cabins that are often exposed to a broad variety of outdoor conditions ranging from temperatures below 0 °C to over 50 °C. On one hand, cabins are typically equipped with heating ventilation and air-conditioning system (HVAC) to create a comfortable environment [14]. On the other hand, the HVAC is usually activated right after entering the cabin and its response time is insufficient to satisfy passengers’ needs and expectations immediately. Hence, the seats may be equipped with contact heating and ventilation systems to compensate rapidly for the extreme conditions and to help overcome adverse effects of excessive heating or cooling of the body through accumulated heat in the seat causing thermal discomfort. Such seat constructions have to be tested in the process of their development with regards to thermal comfort in human studies. Because of high cost and time requirements of human trials, there is a substantial effort to simulate human thermal perception virtually [15–17]. For this reason, numerous virtual manikins or reference models of human anthropometry were developed to reproduce average observed characteristics of a human for human thermal physiology simulations. The most frequently referenced models are the 65MN model by Tanabe et al. [18], the Berkeley thermoregulation and comfort model [19,20], and several Fiala-based models—ThermoSEM by Kingma [21], FPCm by ErgonSim [22], Fiala-FE by Theseus FE [23], FMTK by Pokorny et al. [24], and Human Thermal Module in TAITherm by Thermoanalytics Inc., Michigan, USA [25].

The virtual manikins are usually composed of basic geometric solids, such as cylinders or spheres, representing individual body parts. This division is generally dictated by the resolution needed for proper simulation of human thermoregulation in conjunction with clothing, e.g., changes of skin temperature during vasomotory response, sweating patterns, or typical clothing body coverage. In addition, some models are further divided into sectors facing environment or neighboring body parts, such as anterior, posterior, inferior and/or superior sectors. Such fine resolution of body segmentation allows a much more precise determination of the environmental influence, for instance, projected area factors for detailed direct and diffuse solar radiation and longwave radiation analysis. The summary of the parameters of individual models is presented in Table 1. Despite the clearly defined segmentation of the virtual manikins, it is not clear if the surface area of the assumed sectors in contact with the seat correspond to the seat contact area of a human with the equivalent anthropometry (e.g. height, weight). This is especially critical for the identification of the contact body parts and their surface area involved in seat heating or ventilating. Moreover, skin sensitivity to a thermal stimulus is neither homogeneous over the surface of a human body, nor is the distribution of sweating [26–29]. As a result, there is a need to specify the total seat contact area with a resolution of at least two major body parts—seat and back, both having distinct physiological and perceptual responses.

**Table 1:** Examples of virtual manikins.

Source	Body parts/ body sectors	Sex	Weight (kg)	Height (m)	Total skin area (m^2^)
FPCm [22]	13/41	Unisex	71.4	1.70	1.83
65MN [18]	10/-	Male	74.4	-	1.87
ThermoSEM [21]	10/22	Unisex	73.4	1.73	1.85
Berkeley thermoregul. and comfort model [19,20]	10/-	Female	-	-	1.47
FMTK [24]	12/32	Unisex	73,5	1.71	1.85
Human Thermal Module in TAITherm* [25]‡	13/41	Male	78.6	1.76	1.95

Pair body parts are counted as one.

*50^th^ percentile male body was selected;

^‡^Help desk, ThermoAnalytics Inc., Michigan, USA.

To the best of our knowledge, there is no standard methodology for determining contact area between the body and the seat, and a variety of different methods has been applied. Park et al. carried out a study on seat pressure distribution and preferred driving position of Korean drivers wearing tightly fitting clothing [3]. This was done using dedicated seat pressure blankets with resolution of eight and nine segments on the seat and back, respectively. The test subjects consisted of 10 males and 10 females in body weight groups of: < 59 kg, 60 to 79 kg, >80 kg. Each weight group was evaluated individually, however, there were missing details about subjects’ heights and mean, maximal, and minimal body weights in each weight category.

Another method to determine seat contact area is to draw a border line around a seated manikin on paper placed on the seat [30,31]. The first manikin study employed thermal manikin Fred (height of 1.79 m, body surface area of 1.8 m^2^, unknown weight) placed on various office chairs. In the second study, an Asian Newton type thermal manikin (height and weight of 1.70 m and 65 kg) was employed to examine contact area on airplane seats [31].

In some cases the method was not explicitly stated and the seat contact area was stated as aside information, e.g. study by Oi et al. [28], who presented the seat contact area based on a pool of eight Japanese males sitting on an automotive seat. The group of volunteers was rather homogenous with mean body weight of 57.2 kg (SD ± 3.2 kg) and height of 1.75 m (SD ± 0.01 m). Clothing in the study comprised of long pants, a long sleeved shirt, and a light jacket.

To sum up, the anthropometric data for human subjects was often presented in a narrow range or there were even missing details about subjects’ body weight or height, which makes it impossible to compare various literature sources using objective parameters. In addition, the use of thermal manikins on seats seems not to be representative of human contact area because of their low body weight, unrealistic weight distribution, a rigid body surface without local skin resilience, and no spinal flexibility. Another parameter that is often missing in literature is the precise description of the examined seat and subjects’ clothing. Studies by McCullough [30] and Wu et al. [31] have demonstrated positive correlation between the seat contact area and thermal insulation of clothing. However, the practical use of these results is limited, as they both used thermal manikins.

Another possibility would be to use commercially available seat testers that are dedicated for realistic testing with respect to weight distribution and geometry of the contact parts, e.g. manikin STAN (Thermetrics, Seattle, WA, USA) [32]. However, contact areas examined with this device have not been found in the scientific literature. All the above mentioned issues prevented reliable utilization of literature data in thermophysiological simulations.

The aim of this study was to develop a model describing the body contact area with the automotive seat in relation to body characteristics, such as body weight and height. Human subjects and the Western type Newton thermal manikin were seated on a serial-production automotive seat and the contact area was examined separately for the seat and the back using a modified method based on the study by Park et al. [3]. Finally, the results were compared to the available literature data for both humans and thermal manikins. Since the contact area is crucial not only for accurate manikin measurement of thermal effects of seats, but also necessary for setting up the boundary conditions in simulations using human thermoregulation and thermal perception models, we have also addressed the body resolution of humanoids used in such models. The findings from this study are applicable in clothing, indoor, and transportation research.

## Methodology

### Measurement principle

The methods comprise experimental determination of the seat contact area of human subjects as well as a thermal manikin. The determination of seat contact area for this study was based on printing of a human or manikin silhouette on paper placed on an automotive seat (see sections *Seat* and *Protocol* for more detailed characterisation). The method is proposed because of its relative simplicity and no need for special equipment. In addition, a higher resolution of this approach is expected compared to the circumscribing method, especially on inaccessible parts for circumscribing, e.g. lower back and pelvis area.

After the printing, the paper prints were immediately laid flat and photographed using a 50 mm lens from a distance of 1.3 m. The area of seat print images were determined using graphical software CorelDRAW X8 (Corel Corporation, Ottawa, Canada) by manual tracking of the print borders. The number of the print pixels was compared to the number of pixels of a known reference area in the picture. Additionally, the actual prints were divided into two weight groups split by the median of the sample (80 kg) to visualize potential differences in the shapes of the seat prints depending on the subjects’ weight.

### Human subjects

**Fig 1:**
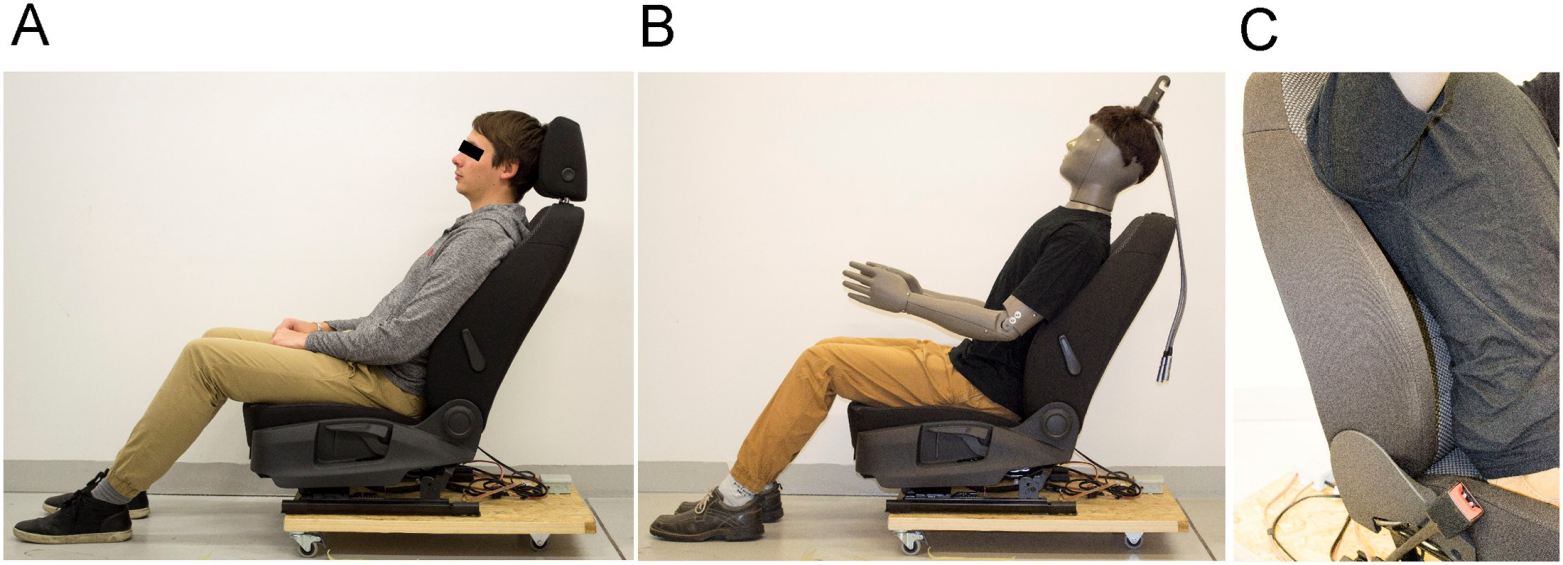
Illustration of the seating position. A human subject (A), the Newton manikin (B), and the detail of insufficient contact of the manikin’s lower back with the back rest (C).

The research was approved by Ethical committee at Department of Biomedical Engineering at Brno University of Technology (document EK:01/2018, approved on 5. January 2018) and all 13 subjects (12 males and 1 female) agreed to enter the experiment voluntarily signing a written informed consent. The subjects cover a majority of European population (approximately 82%) in terms of body mass index (BMI) ranging from the lower limit of normal weight (18.5 kg/m^2^) to the upper limit of overweight (29.4 kg/m^2^) [33], and represented the white ethnic group with average characteristics: body weight of 80.6 kg (SD 11.7 kg), height of 1.80 m (SD 0.05 m), body surface area of 1.99 m^2^ (SD 0.02 m^2^) calculated according to the Du Bois equation [34], BMI of 24.8 kg.m^-2^ (SD 3.0 kg.m^-2^), and age of 30.5 (SD 3.4 years). The participants were instructed to wear a pair of long cotton pants, underwear, and T-shirt with normal fit (Fig 1A). The individual in Fig 1A has given written informed consent (as outlined in PLOS consent form) to publish these case details.

### Thermal manikin

The Newton type thermal manikin (Thermetrics, WA Seattle, Western type manikin) was employed for comparison of the contact area with the one measured by human subjects using the same method. The manikin body size corresponds to a 1.79 m tall western male. The construction of the manikin is intentionally lightweight (27.5 kg), thus, having lower thermal inertia and allowing easier manipulation in its typical application in clothing research. Here, it is necessary to easily dress, undress, and set the manikin in the desired position. For this reason, the manikin is also equipped with ten movable joints (ankles, knees, hips, elbows, shoulders). However, it does not allow spinal flexing and its surface is rigid (Fig 1B).

### Seat

**Fig 2:**
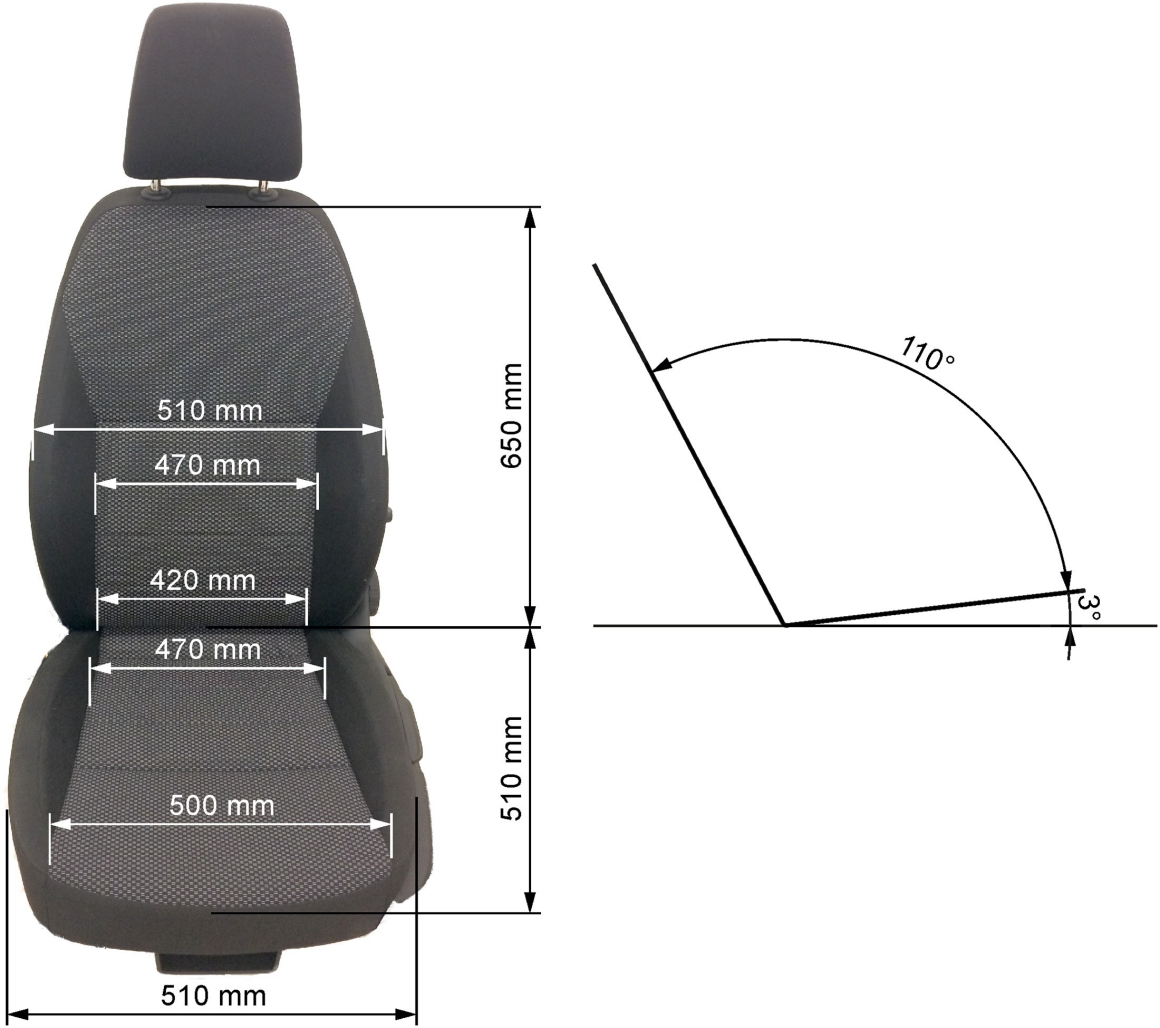
Projected dimensions of the seat used in this study.

The seat used for the study represented a serial-production front seat of a middle class passenger car. The construction of the seat consists of metal chassis, polyurethane cushioning, and polyester upholstery. The thicknesses of the seat and back rest cushioning are approximately 5.5 and 4.5 cm, respectively, and the participants rated the stiffness of the seat as medium (scale: *soft*, *medium*, *hard*). The setup of the seat was fixed in all trials (details in Figs 1 and 2) with the adjustable lumbar support set to maximum. Height of the front lip of the seat was approximately 37 cm above the floor. The trunk-thigh angle was set to 110° based on the preferred driving posture of a bodyweight group ranging from 60 to 79 kg [3].

### Protocol

The method to determine the seat contact area separately for the buttocks and the back was firstly validated using water based paint applied to a plastic foil attached to the back, buttocks, and lower thighs of the participant. Next, the person sat down on the seat covered by two separate sheets of paper (specific weight of the paper 80 g.m^-2^) and stood up again after approximately 15 seconds. The subjects were asked to sit in a comfortable position, but without slouching and with fully rested thighs on the seat (Fig 1). The print of the contact area of the body on the paper padding of the seat was then used for further processing. Finally, the paint was replaced by spraying water directly on the participants’ clothing. The aim of this was not to soak the clothing, but to create a thin film of water droplets on the clothing that was later transferred to the paper with mitigated effects of lateral wicking. The specific weight of the paper used in this study was approximately 40 g.m^-2^ to improve uptake of the water, and hence, leaving a clear print border. The finer paper is also capable of copying the seat surface deformations without major buckling. These changes were sufficient to identify the contact area on the paper with better time efficiency and no contamination hazards. The contact area of the Western type manikin was determined using the same method and clothing (all garments size M). In this case, the head rest of the seat was removed to allow the manikin to support its back on the seat, since the neck does not flex (Fig 1B).

### Cross-comparison with literature data

In the final step, the experimental results were compared to the findings from literature. This was done to assess differences among data from the human, manikin, and virtual manikin studies using various approaches and the possibility to use virtual thermal manikins for simulations with a seat contact. Despite not having the realistic contact areas from the seat tester STAN, the surface areas of its active parts were assumed for the comparison. Next, for the Fiala virtual manikin [22], following segments were selected to stand for contact surfaces: back—the sum of posterior thorax and posterior abdomen, and buttocks—the sum of posterior hips and posterior upper legs. In case of the TAITherm virtual manikin [25], the back contact was selected to consist of back and a half of upper abdomen with assumption of manikin symmetry. Finally, the buttocks contact was assumed to be posterior thighs and a half of lower abdomen. An overview of the cases and selected clothing is shown in Table 2.

**Table 2:** Overview of the cases for cross-comparison with the literature data.

Study	Subjects	Ethincs	Seat	Clothing	Method
This study	12 M; 1F	White	Automotive	Normal fit—T-shirts, Trousers	Printing a silhoutte
Park et al., 2015	10 M; 10 F	Korean	Automotive	Tightly fitting outfit	Pressure sensitive blankets
Oi et al., 2012	8 M	Japanese	Automotive	Shirt, Jacket, Trousers	Unknown
Wu et al., 2016;	Asian Newton	Asian	Airplane	Shorts, T-shirt	Circumscribing
McCullough, 1994	Manikin Fred	Unknown	Executive chair	Trousers, Shirt	Circumscribing
This study	Western Newton	White	Automotive	Normal fit—T-shirts, Trousers	Printing a silhoutte
Fiala virtual man. [22]	Unisex	Global	-	Nude	Posterior parts
TAITherm virtual man. [25]	Male	White	-	Nude	Posterior parts
STAN seat tester ‡	Male	White	-	Nude	Active areas of the manikin

M-males, F-females.

^‡^Help desk, ThermoAnalytics Inc., Michigan, USA.

### Model description and statistical analysis

The collected data was evaluated in the Microsoft Excel (Microsoft Corporation, Redmond, WA, USA) and the linear regression model was proposed to fit the data from the seat prints with predictors, namely: weight, height, body surface area, and BMI. This was done despite the non-linear behaviour of the seat and body deformation that affects resulting contact area. However, in the examined range of the predictors (see section *Human subjects* for the details), linearisation of the interaction of the human body with the seat is expected to have strong correlation and sufficient precision for the given application in thermophysiology. Further, both body surface area and BMI are power functions of weight and height, but both were included in this study as additional parameters because of their frequent occurrence in literature and praxis. Finally, selected cases from literature (Table 2) were added to the comparison, wherever applicable, with respect to the scope of the published parameters.

Next, the coefficient of determination *R*^*2*^ was calculated to express the proportion of the variation in the dependent variable that is predictable from the independent variable. Further, the root mean square deviations *RMSD* between the measured data and predictions were expressed to assess the accuracy of the models. Finally, two intervals were defined for the linear regression models. Firstly, a confidence interval for the best-fit line for the collected population with 95% confidence level was plotted. This allowed visualisation of margins for the slope and intercept for each regression model. Secondly, prediction intervals were calculated to estimate an interval in which future individual observations of contact area will fall with 95% probability [35].

## Results

**Fig 3:**
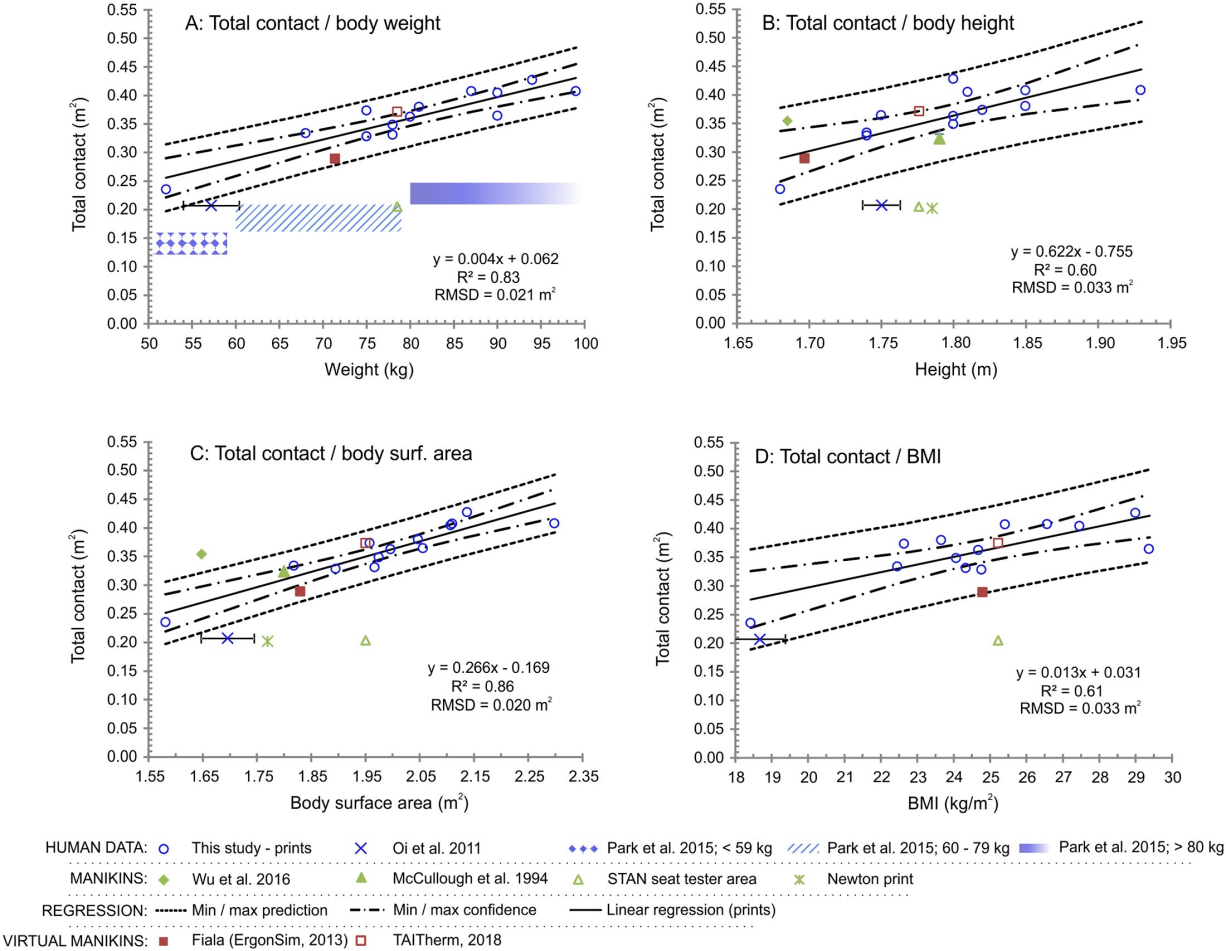
Total seat contact area. The total seat contact area dependent on body weight (A), body height (B), total body surface area (C), and Body Mass Index (D).

**Fig 4:**
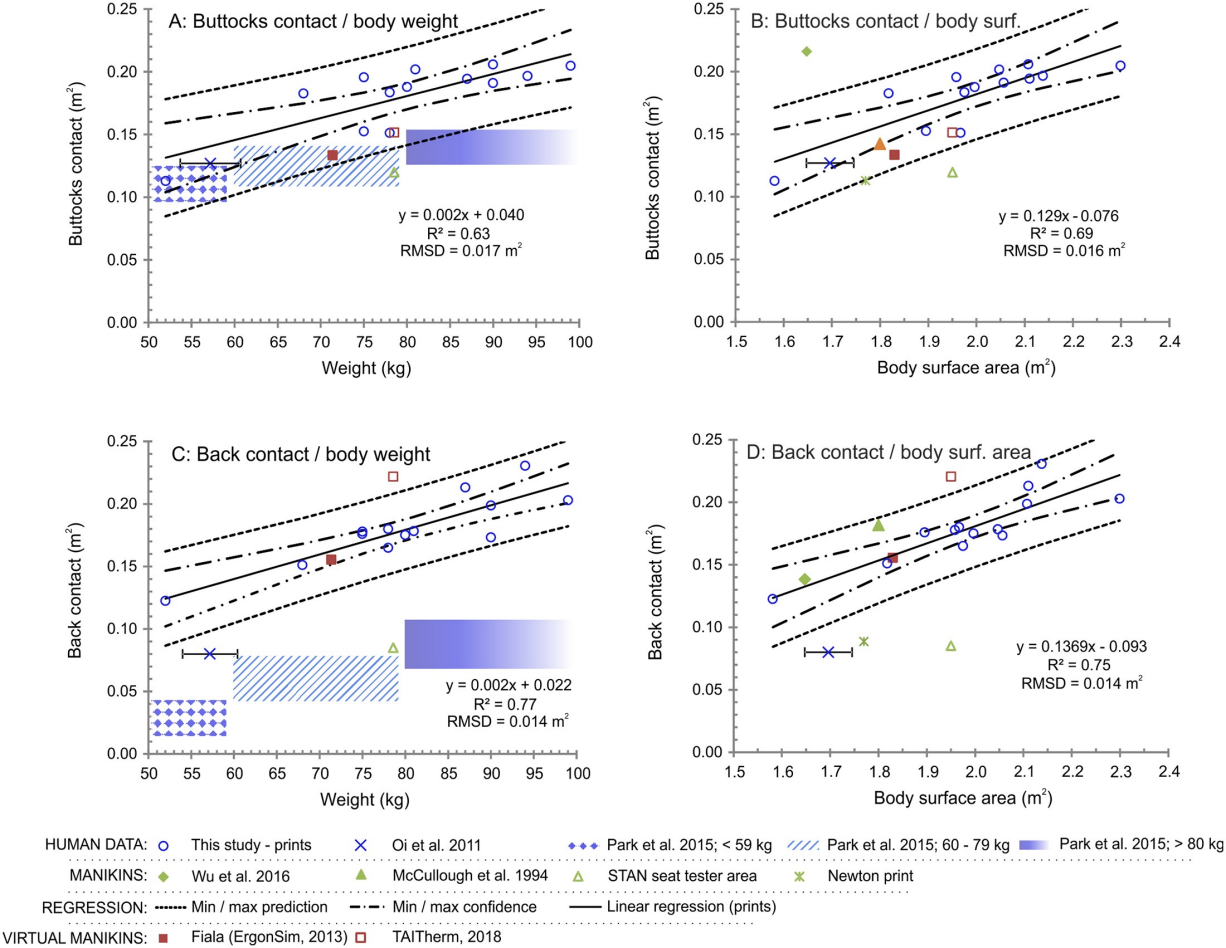
Local body contact areas. Contact areas at back and seat dependent on body weight (A and C), and total body surface area (B and D), respectively.

Fig 3 relates total seat contact area to two basic anthropometric measures, body weight and height, and two derived measures, total body surface area and BMI. The average total seat contact area is 18% of the body surface area. Fig 4 shows the predictive functions of the seat and back contact area separately in relation to body weight and body surface area being selected on the basis of the highest *R*^*2*^ values and the lowest values of *RMSD*. The points marked with violet colour stand for human studies, whereas green and red markers represent manikin and virtual manikin studies, respectively. Error bars indicate the standard deviation if reported in the original source. A special case is the study by Park et al. (2015), in which neither the actual mean values of each bodyweight group were presented, nor the upper and lower weight limits. Thus, we interpreted the results from the study as areas (Figs 3A, 4A and 4C). Moreover, 95% confidence intervals were plotted for each linear regression model with dotted lines, and the dashed lines depict prediction intervals covering 95% of the population (Figs 3 and 4). The complete dataset on which this publication is based, is provided in the supporting information file S1 Dataset.

## Discussion

### Total contact area predictions

Body weight and total skin area were identified as the best predictors for the total contact area. The values of *R*^*2*^ ranged from approximately 0.60 (body height, BMI) to 0.86 (body weight, body surface area). Correspondingly, the values of *RMSD* were of 57% lower if body weight and body surface area were used as predictors compared to body height and BMI. This indicates that changes in body weight or body surface area have a greater impact on the changes in total contact area than height or BMI within the scope of this study.

**Fig 5:**
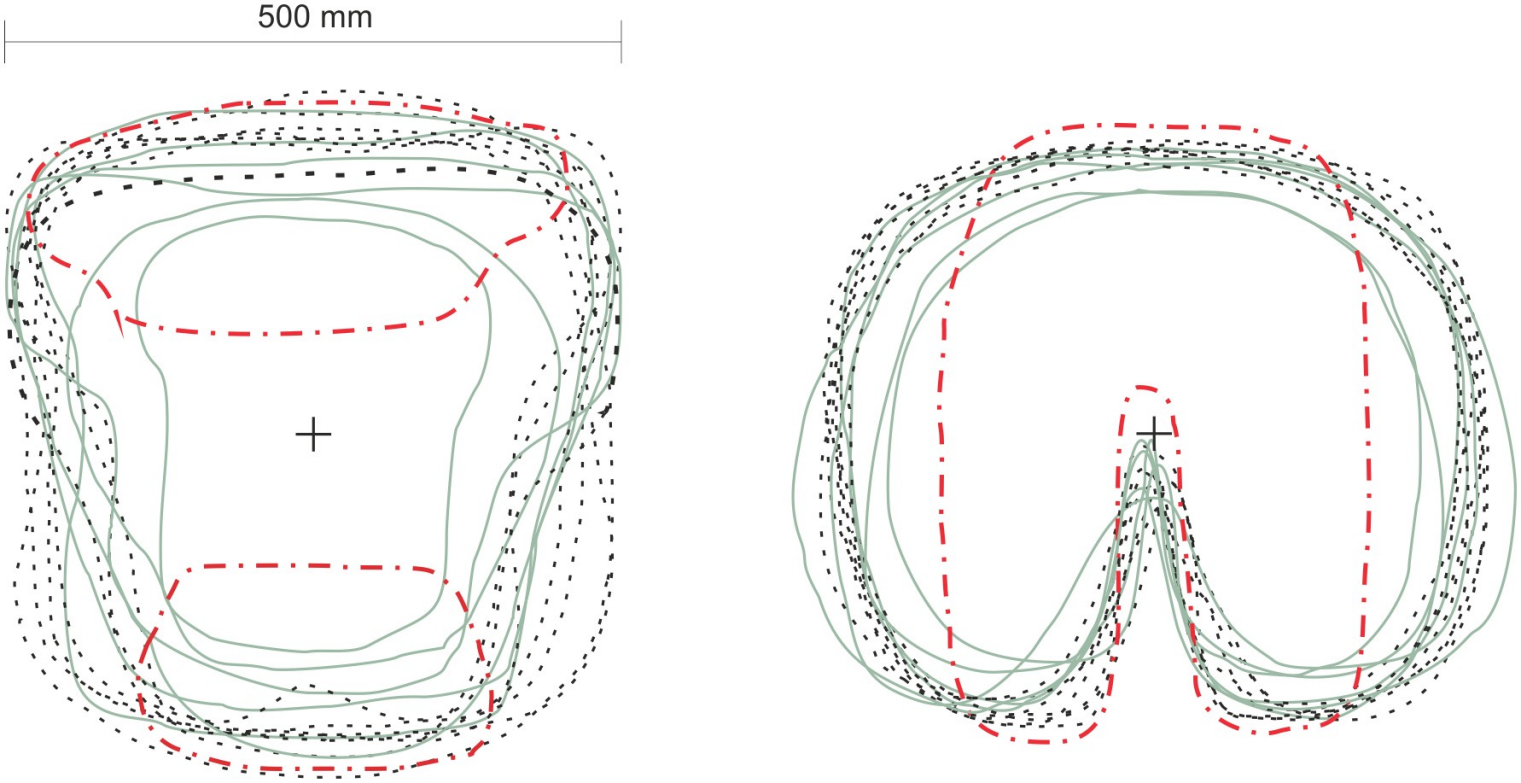
Centred shapes of the seat prints. Back prints (left) and buttocks prints (right). Dashed line indicates a subjects group with body weight of more than 80 kg, continuous line with less than 80kg, and red dotted line depicts the contact of the Western Newton type manikin.

Next, if we intuitively presume that the height has a dominant effect on the print length and the weight on the print width, a partial support for this statement could be found in Fig 5. The back prints exhibit greatest standard deviation in the lumbar width (SD 0.08 m, min/max width 0.24/0.49 m), as opposed to the back length (SD 0.06 m, min/max length 0.36/0.57 m). Similarly, the variability of the seat print width is greater (SD 0.05 m, min/max width 0.43/0.61 m) than the seat print length (SD 0.03 m, min/max length 0.37/0.48 m). On the other hand, since the seat width is 0.51 m and several prints exceeded this size, we can also assume that higher weight contributes to a greater imprint of a person into the seat, thus, leaving a larger print area in all directions.

Secondly, total body surface area calculated according the Du Bois equation has the highest explanatory power as opposed to the BMI. In both skin area and BMI formulas, the weight has a lower power than the height, but the weight has a greater impact on the result in a range of meaningful values for adult seat occupants, e.g. from 45 to 120 kg. These values were calculated using the limits for underweight and overweight (from 18 to 30 kg/m^2^) and a likely range of the occupants’ body height (from 1.6 to 2.0 m). Most importantly, the interpretations of the formulas are different. The BMI can be explained as a screening tool for indication of high body fat content, calculated as weight over square of height, and its nature has been criticized for not capturing the distribution and proportion of lean mass and body fat [36]. This is likely the cause for the relatively low *R*^*2*^ of 0.61 if compared to the straightforward parameter, such as the skin surface area (*R*^*2*^ = 0.86).

### Local contact area predictions

The models for the local contact surface with the buttocks and back were based on the body weight and body surface area as they showed the best predicting capabilities out of the examined parameters. In both cases, the models for back contact area have smaller values of RMSD and higher *R*^*2*^ compared to the seat area models, in other words, the models for back are more accurate.

In Fig 5, the prints for seat and back are divided into two weight groups split by median of the sample (80 kg). The shoulder width remains in the majority of cases almost identical and is outlined by width of the seat, whereas, in most of the cases under 80 kg, the lumbar contact has narrower contours (Fig 5 left). This shaping reflects the distribution of muscles and body fat with increasing weight, where the larger fat deposits occur in the lumbar region [37]. On the other hand, the differences in the print shape and size are less pronounced in case of the buttocks (Fig 5 right).

Finally, literature on an apparel design discusses several predominant male and female body types based on the proportions of hips-waist-back dimensions, lengths of the limbs and crotch [37,38]. The variability of the body shapes is also projected into the seat prints, where a contact area of two subjects with a similar anthropometric measure is not identical. For instance, in Fig 5 left, the widest seat print belongs to a person weighing less than 80 kg with muscular thighs that leave wider print than ones of heavier subjects with less developed musculature at thighs. This also explains differences between lower and upper prediction lines that are, in the best-case scenarios, of around 0.10 m^2^ for total contact (Fig 3C), 0.07 m^2^ for seat (Fig 4B) and 0.06 m^2^ for the back (Fig 4C).

### Cross-comparison with literature data

The findings from this study were compared to two human studies, two manikin studies, and two virtual manikin geometries. The first human study by Oi et al. [28] yields the total contact area of 33% lower compared to the mean prediction of this study. Yet, these values are on the edge of the prediction intervals unless the height was used as a predictor (Fig 3). Next, the seat contact area is below the mean predictions too, but within the confidence and prediction intervals (Fig 4A and 4B). The situation is different on the back, where the local contact is of 68% lower compared to mean predictions and out of prediction intervals in all cases (Fig 4C and 4D). The consistency of the seat contact and mismatch of the back contact areas point out potential differences in the seat involved in the study that was described only as an automotive seat. Secondly, the discrepancies might be caused by differences in body shapes between the Japanese and white ethnicities. Possible explanation of this hypothesis may be found in the Nakanishi and Nethery [39] who examined differences in anthropometry of Japanese and white-American university male students. Significantly lower girths in Japanese at all body parts that build a contact with a seat were found, but no significant differences in percentage of body fat. Wang et al. [40] carried out a study on differences between Asians and white males and females in range from 18 to 94 years. The main findings show the Asians having lower BMI, while having an increased amount of adipose tissues than the white of both sexes. This can be explained by a different muscle constitution between the ethnicities that may be the cause for significant differences in the back contact area, and less pronounced differences in the seat contact.

The seat contact areas from the second human study by Park et al. [3] were presented in three body weight categories and the results are again below predictions of this study (Figs 3A, 4A and 4C). The mean contact area on the back for 70 kg person is substantially lower of about 0.1 m^2^ compared to predictions from this study (difference of 169%). On the other hand, the seat contact area intersects with the lower prediction intervals (Fig 4A). The first likely explanation for the mismatch may stem from the focus on driving conditions by which the contact area distribution is asymmetric and variable, thus, being lower than that of a rested person. The decrease of the contact is caused by the use of the right leg and arms to control the vehicle [3]. Secondly, the subjects in the study were Korean with differences in the anthropometry when compared to the white [40]. Thirdly, the contact area was examined by pressure sensitive blankets with a resolution of 1296 sensing points, and size of 45 × 45 cm, whereas the print sizes in this study exceeded 45 cm in the majority of cases. Therefore, the blankets might not have been sufficient to cover all possible contact areas, especially on the back and in high body weights, where the dimensions of the prints are the greatest (Fig 4C).

Next, data from both Asian, Western Newton type manikins, and the seat tester STAN shows great discrepancies (Figs 3 and 4). The STAN has its contact area below predictions, however, this is expected since the assumed contact areas are not actual prints, but active sensing areas of the device. The seat contact area of the Asian type manikin is greater by 58% compared to the predicted mean from this study and is out of the prediction interval, whereas the back contact area matches the confidence and predictions intervals. Admittedly, the ratio between seat and back was not presented in the original paper and was adopted from literature being equal to 60:40 [28]. Yet, this does not have an impact on the total contact area that is still relatively high compared to the predictions (Fig 3C). The disagreement may stem from a different way of determining the contact area. This was neither explained in detail, nor was it explained how the contact area on inaccessible parts was identified (e.g., lower back and hips). For instance, authors may have taken into consideration the contact border line wherever the clothing touches the seat. In this study, the contact was ascribed to areas determined by both applying a pressure and moisture from the clothing. Additionally, the authors have not explained how they adjusted the manikin parameters given by the manufacturer, such as 1.69 m and 27.5 kg of manikin body height and weight, respectively [41]. The additional weight and its distribution were not explained in the paper and may have contributed to a larger seat contact. Finally, the examined seat was an airplane seat with unspecified dimensions and setup.

The Western type manikin, used in this study, has smaller seat and back contact areas of 35% and 69%, respectively, if compared to the mean human predictions. This is rather anticipated with regards to the rigid and lightweight manikin body construction (27.5 kg for manikin as compared to approximately 65 kg human of a similar anthropometry), and consequently, lower print area. In reality the manikin touches the back rest only at the upper back and hip level with an air gap covering the whole lumbar area. These findings are supported by Fig 1C, where the gap is photographed, and also by the shape of the manikin prints in Fig 5 (red dotted line).

Another manikin study presented by McCullough [30] shows a good match with the total and local predicted values. In this case the weight of the manikin is unknown, but with respect to the year 1994 when the paper was published, the manikin could have been made of metal with consequently higher construction weight. This might imply more realistic immersion to the seat, resulting in the larger contact area with the seat. Further, the manikin might have had different curvature on the back that allows better contact with the seat. The seat involved in the study was executive chair with a similar shape to the car seat.

The total and local contact areas of the Fiala virtual manikin match the predictions well and are in all cases within the prediction lines (Figs 3 and 4). Such findings are favourable to the further utilization of the virtual manikin in the simulation of the local effects of the seat on the thermo-physiology and human thermal perception. The TAITherm manikin has similarly good match with human observations in case of the total contact and buttocks, whereas the back contact area is above prediction lines.

Virtual manikins with verified contact area could be effectively used to predict effects of seat heating or ventilation on a human. On the other hand, despite the match of the proposed contact parts of the virtual humanoid and human contact area, it is still questionable, whether the humanoid geometry is adequate for the seat simulations. Today, the humanoids used in thermophysiological models are usually composed of basic solids, e.g. cylinders (Table 1), whereas the shape of the back contact is rather conical in majority of cases (Fig 5). Greater contact area is thus at the upper back rather than on its lower part. Insight into the seat contact opens up opportunities to refine the segmentation on contact parts with respect to different level of sensitivity to hot and cold stimuli [27] and distribution of sweat [29].

### Practical implications

As discussed in section *Local contact area predictions*, the differences between the lower and upper prediction lines are not negligible. This may have further impact on practical applications of the results, such as additional thermal insulation provided by the seat, seat heating or ventilation, etc. To demonstrate this, by example, we considered a seat heating of 268 W.m^-2^ [28] and uniformly distributed power over the surface of the seat. The total contact heating power for the virtual manikin anthropometry would be 77 W. For a human with the same skin area, the range of the heating power is between 72 and 98 W. Such variation should be accounted for in representation of simulation results and it needs to be explored further what would be the influence of this variability on the human thermo-physiological response in a specific application.

The study captures the range of predominant European weight in terms of BMI (Details in section *Human subjects*) and the extremes were not examined. With regards to finite dimensions of the seat, it is expected that the upper limit of contact area exists as the seat gets filled with the body with increasing BMI. Therefore, an extrapolation of the results, especially in direction of higher body weights, might not be valid.

Another implication of the findings is in utilisation of thermal manikins in measurement of seat thermal properties. This study shows that different manikins and approaches of determining seat contact area yield different results. Therefore, it is advisable to ensure similar contact of the manikin to the one of humans before performing any measurements, e.g. by applying an additional weight to the figurine.

### Limitations and sources of error in the experimental work

The resolution of the proposed method is limited by wicking and soaking of the water into the paper. However, the amount of applied water was controlled and kept low to eliminate these effects. Next, the seat print borderline was circumscribed manually in graphical software (Details in section *Protocol*), therefore, the borderline might not be copied perfectly. However, the contribution of these two sources of error is expected to be negligible compared to the variations in human data.

Finally, the sample of participants is relatively low, but it corresponds to the aim of this study to provide an overview into the problematics of seat contact area with regards to thermophysiological simulations, which was not as clear as to present. A consistent and robust method to carry out contact area measurements for any population and seat of interest was proposed. This allowed us to discuss and compare various approaches from the literature sources in a broader context.

## Conclusions

To conclude, linearity between the contact area and body weight, height, skin surface area, and BMI was found within the scope of this study. The most reliable and precise predictors for the contact areas are the body weight and the body surface area. Next, the numerical results are valid for the white ethnicity and might not be applicable in other ones, e.g. in the Asians because of differences in body constitution. Further, the cross-comparison shows inconsistencies in determination of the contact area using thermal manikins. The reasons for this are possible differences in methodologies, manikins’ rigid structure, low body weight, and no spinal flexibility of the manikins. In case of the virtual manikin used in the human thermoregulation model by Fiala, it was verified that posterior thorax, abdomen, hips, and upper legs can be used as representatives of the seat contact area.

## Supporting information

S1 Dataset Summary of the data for the publication. (XLSX)
